# Finding synchrony in the desynchronized EEG: the history and interpretation of gamma rhythms

**DOI:** 10.3389/fnint.2013.00058

**Published:** 2013-08-12

**Authors:** Omar J. Ahmed, Sydney S. Cash

**Affiliations:** ^1^Department of Neuroscience, Brown UniversityProvidence, RI, USA; ^2^Department of Neurology, Massachusetts General Hospital, Harvard Medical SchoolBoston, MA, USA

**Keywords:** desynchronized EEG, gamma oscillations, synchrony, history of gamma rhythms

## Abstract

Neocortical gamma (30–80 Hz) rhythms correlate with attention, movement and perception and are often disrupted in neurological and psychiatric disorders. Gamma primarily occurs during alert brain states characterized by the so-called “desynchronized” EEG. Is this because gamma rhythms are devoid of synchrony? In this review we take a historical approach to answering this question. Richard Caton and Adolf Beck were the first to report the rhythmic voltage fluctuations in the animal brain. They were limited by the poor amplification of their early galvanometers. Thus when they presented light or other stimuli, they observed a disappearance of the large resting oscillations. Several groups have since shown that visual stimuli lead to low amplitude gamma rhythms and that groups of neurons in the visual cortices fire together during individual gamma cycles. This synchronous firing can more strongly drive downstream neurons. We discuss how gamma-band synchrony can support ongoing communication between brain regions, and highlight an important fact: there is at least local neuronal synchrony during gamma rhythms. Thus, it is best to refer to the low amplitude, high frequency EEG as an “activated”, not “desynchronized”, EEG.

## Richard Caton: the first EEG

It seems appropriate that a review of the history of rhythms should begin in the city of Liverpool. Long before the guitars, drums and microphones of the Beatles, there was the galvanometer of Richard Caton, a local physician who went on to become Lord Mayor of Liverpool. In 1875, Caton published what is thought to be the first account of the oscillatory activity of the brain (Caton, 1875; Brazier, 1961; Niedermeyer and Lopes da Silva, 2005). His description of recordings made from rabbits, cats and monkeys was all of nine sentences long. The 5th sentence read (Caton, 1875):

Feeble currents of varying direction pass through the multiplier when the electrodes are placed on two points of the external surface, or one electrode on the gray matter, and one on the surface of the skull.

Caton elaborated on his observations 2 years later (Caton, 1877):

The current is usually in constant fluctuation; the oscillation of the index being generally small … At other times, great fluctuations are observed, which in some instances coincide with some muscular movements or change in the animal's mental condition.

Caton went further. He attempted to explore the functional correlates of these electric currents and found that shining a bright light on the retina led to “variations in currents” in the “posterior and lateral parts” of the brain (Caton, 1877). Thus Caton not only documented the first electroencephalographic (EEG) recordings, but he was also among the first to report sensory evoked potentials in the brain.

Caton's work built upon the observations of Luigi Galvani, who, in 1791, used the notion of “animal electricity” to explain why frog leg muscles twitched in response to sciatic nerve stimulation (Galvani, 1791; Swartz and Goldensohn, 1998). The “multiplier” mentioned in Caton's papers was a reflecting galvanometer, named after Galvani, but invented by Johann Schweigger and improved by William Thompson (Durgin, 1912). Caton used non-polarizable electrodes similar to those employed by Emil du Bois-Reymond, who had used them to show electrical “negative variations” (action potentials) in active peripheral nerves (Brazier, 1961; Niedermeyer and Lopes da Silva, 2005). In 1870, Fritsch and Hitzig discovered that the brain could be electrically stimulated to evoke movements (O'Leary et al., 1976). David Ferrier built upon this work by stimulating the cortex of monkeys (Ferrier, 1876; Niedermeyer and Lopes da Silva, 2005), and Caton explicitly cited the influence of Ferrier's thinking on his work (Brazier, 1961). Of course, none of this electrophysiological work would have been possible without the pioneering work of Giovanni Aldini, Alessandro Volta, Georg Ohm and others in understanding the basic principles of electricity (Durgin, 1912).

## Adolf Beck: the desynchronized EEG

In 1891, Polish physiologist Adolf Beck, without knowledge of his predecessor's work, reproduced many of Caton's results, observing “continuous waxing and waning variation(s)” in electrical recordings from the brains of rabbits and dogs (Beck, 1891; Brazier, 1961). Beck, however, made a new, important observation relating to these oscillations (Beck, 1891; Brazier, 1961):

In addition to the increase in the original deviation during stimulation of the eye with light, rhythmic oscillations … disappeared. However this phenomenon was not the consequence of light stimulation specifically for it appeared with every kind of stimulation of other afferent nerves.

Thus Beck provided the first account of the so-called desynchronized EEG. This desynchronized activity was characterized by the “disappearance” of oscillations during arousal or sensory stimulation.

Was this desynchronized state truly devoid of any rhythmic oscillations? Or, as Mircea Steriade was to put it over a century later, was the desynchronization “more apparent than real” (Steriade, 1996)? Even if there were systematic oscillations during the desynchronized state, one thing was obvious: their amplitude was significantly decreased compared to those observed in the resting state. Technical improvements of the recording equipment were thus a prerequisite to answering these questions.

## Vladimir Neminsky and Napoleon Cybulski: the frequency of oscillations

The electrophysiologists of the 19th century were limited by the poor amplification and limited temporal resolution of their galvanometers, and by the fact that they were not yet able to record and store the oscillatory brain activity they observed. This changed at the turn of the century when, in 1901, Willem Einthoven invented a more sensitive string galvanometer that recorded on photographic paper the shadows created by electrically-induced deflections of a string (Einthoven, 1901; Swartz and Goldensohn, 1998). Einthoven's galvanometer was intended for, and first applied to, recordings of heart electrocardiograms, but it would eventually be used by Vladimir Neminsky to study the frequencies of EEG rhythms. In 1913, Neminsky published the first paper to include photographic EEG reproductions (Pravdich-Neminsky, 1913). In the same paper, he described the frequency of EEG oscillations (Pravdich-Neminsky, 1913; Brazier, 1961):

“Spontaneous” fluctuations led from the surface of the brain (and from the dura mater) varied fairly significantly from 12 to 20 up to approximately 35 per second.

He also noted that the frequency of these oscillations fell to 4–7 per second during asphyxia (Brazier, 1961). In 1914, Napoleon Cybulski, Beck's mentor, reported the results of his own experiments using the string galvanometer. Recording from monkeys, he found that the frequency of the “spontaneous” oscillations ranged from 15 to 20 per second, but increased to 18 to 22 per second after peripheral nerve stimulation (Cybulski and Jelenska-Macieszyna, 1914; Brazier, 1961). In the same paper Cybulksi also documented, for the first time, the EEG correlates of epileptic seizures in dogs.

Thus a rich picture of rhythmic activity in the brain was already beginning to emerge. The exact frequency of oscillations was not fixed, but “spontaneously” fluctuating over wide ranges. In addition, Beck, Neminsky and Cybulksi's afferent nerve stimulation experiments suggested that active processing of sensory information was accompanied by reduced amplitude, desynchronized EEG signals. However, these active EEG states could still be rhythmic, and in fact, were sometimes accompanied by an increase in oscillation frequency.

## Hans Berger: the human EEG, alpha and beta rhythms

In the first of his 23 papers published between 1929 and 1938, Hans Berger reported the first EEG recordings from humans (Berger, 1929; Gloor, 1969). Working at the University of Jena, Berger used platinum needle electrodes, attached on one end to the scalp of his 15-year-old son, Klaus (among others), and on the other end to a double-coil Siemen's galvanometer. Berger placed the electrodes at the frontal and occipital poles of the head, thus sampling non-local voltage differences spanning practically the whole brain. Berger reproduced many features seen in the earlier EEG animal recordings. In particular, he observed an 8–12 Hz rhythm while his son sat passively with his eyes closed. Since this was the first, most prominent rhythm he observed, he named it the alpha rhythm. However, in spite of Berger's suggested terminology, this medium-frequency rhythm was referred to as the Berger rhythm for many years to come. When his subjects opened their eyes, Berger noticed a suppression of the alpha rhythm, replaced by a lower amplitude, higher frequency, and 14–30 Hz rhythm. Berger called this the beta rhythm. Berger also replaced Neminsky's hybrid Greek-Latin term “electrocerebrogram” with the entirely Greek name, electroencephalogram (Brazier, 1961). Despite the substantial efforts of those preceding him, Berger is widely credited as being the “Father of Electroencephalography” (O'Leary, 1970), although “Father of *Human* Electroencephalography” is perhaps more appropriate.

## Herbert Jasper: gamma rhythms and the 1/f power-frequency relationship

The first mention of the gamma-rhythm appears to have come in a 1938 paper by Herbert Jasper, then at Brown University, and Howard Andrews (Jasper and Andrews, 1938):

In some of these records this irregularity is due to still higher frequencies present simultaneously with the alpha and beta frequencies … These higher frequencies (from 35 to 45 per second, which might be called gamma waves) have not as yet been observed with sufficient regularity for analysis.

Jasper was also one of the first to explicitly point out a general relationship between EEG amplitude and frequency: “It appears as a general rule that the amplitude of brain potentials decreases with their frequency” (Jasper, 1936). In subsequent years, as Fourier analysis was more commonly applied to EEG signals to generate power spectra, this decrease in amplitude or power with increasing frequency became known as the 1/f relationship (Pritchard, 1992). The underlying neural or artifactual origins of this 1/f relationship are still heavily debated (Buzsáki, 2006; Logothetis et al., 2007; Bedard and Destexhe, 2009).

## Giuseppe Moruzzi and Horace Magoun: the role of the reticular formation

Giuseppe Moruzzi and Horace Magoun originally intended to stimulate the superior cerebellar peduncle to study its inhibitory effects on the motor cortex (Siegel, 2002). However, these fibers ran through the midbrain reticular formation (RF), and Moruzzi and Magoun ended up stimulating cells in the RF (Moruzzi and Magoun, 1949). They found that RF stimulation had profound effects on the EEG of anesthetized cats: there was a clear shift from low frequency high amplitude oscillations to what they called an “activated” EEG (but was previously, and subsequently, referred to as the “desynchronized” EEG). This activated EEG was accompanied by higher frequency oscillations, normally seen in aroused, active waking states. Using increased stimulation strengths they also showed that the high frequency state would persist after the stimulation had stopped. This effect was seen in all cortical regions (Moruzzi and Magoun, 1949), thus suggesting that the RF was responsible for mediating the desynchronized cortical states seen by Beck, Neminsky and Cybulski in response to afferent nerve stimulation. The role of the RF in generating sleep-wake cycles has been explored in detail over the years (Steriade, 1996; Jones, 2008). In particular, it is believed that the generation of “activated” high-frequency states is mediated by projections from the RF to the non-specific nuclei of the thalamus, which then project to a wide range of cortical areas. It is worth pointing out here that the neuromodulator acetylcholine can also induce the desynchronized, high frequency cortical states associated with wakefulness and alertness. Cholinergic projections from the nucleus basalis of the basal forebrain are thought to mediate this effect (Buzsaki and Gage, 1989).

EEG has developed into an invaluable tool in clinical settings (Niedermeyer and Lopes da Silva, 2005), and hundreds of labs have analyzed the behavioral and cognitive correlates of EEG in both humans and laboratory animals using an ever-more sophisticated set of analytical techniques (Fries et al., 2001; Bokil et al., 2006). This has led to significant advances in our understanding of the neuronal underpinnings of oscillations.

## The source of EEG and LFP signals

“Field potentials, although easy to record, are difficult to interpret.”

—Ulla Mitzdorf (Mitzdorf, 1985)

So far, we have used “EEG” as the umbrella term to describe extracellular electrical recordings from the brain. More accurately, the use of the term EEG should perhaps be limited to non-invasive recordings from the scalp, with the term electrocorticogram (ECoG) used to describe recordings made from electrodes placed directly on the cortical surface. Local field potential (LFP) refers to the signal recorded using deeper microelectrodes, located in specific cortical or subcortical layers. LFP signals are typically an order of magnitude larger than their EEG counterparts. This is because the currents are attenuated as they pass through various cortical layers and through the entire thickness of the skull before being recorded as EEG signals, a disparity that had been noted by both Caton and Beck (Caton, 1877; Beck, 1891; Brazier, 1961).

It is generally believed that the LFP signal reflects the electrical currents associated with synaptic activity in a local population of neurons around the electrode (Renshaw et al., 1940; Mitzdorf, 1985, 1991; Niedermeyer and Lopes da Silva, 2005). An excitatory synaptic input generates a sink, as positively charged ions leave the extracellular space and flow into the cell. This should result in a negative deflection of the LFP on an electrode located near the synapse. Similarly, an inhibitory post-synaptic potential should lead to a source, resulting in a positive deflection of the LFP. However, shunting inhibition should result in zero net current flow, and should therefore be associated with no changes in the LFP.

This, however, is a gross oversimplification, and the situation becomes very complicated very quickly as additional details are considered. Each synaptic sink is accompanied by sources at other points on the neuron, as positive ions flow down the dendrites, cell body and axon and eventually exit the cell as leak conductances. Thus the exact location of the electrode with respect to the synapse will determine the magnitude and polarity of the associated LFP deflection. The presence of multiple excitatory and inhibitory synapses along the extent of a pyramidal cell presents another confound. If multiple synapses are activated at different points in time and space, the resulting distribution of sinks and sources becomes difficult to predict, and is likely to underestimate the excitatory activity due to various cancellation effects between the positive and negative current flows around the synapses. The presence of additional cells in the vicinity of the electrode, each with their own unique morphology, synapses, sinks and sources increases the complexity dramatically.

## Neurons fire synchronously during LFP rhythms, even fast rhythms

In the face of this overwhelming geometric and electrical complexity around an electrode, the LFP and EEG signals still reveal systematic oscillations. How is this possible? This brings us to the importance of synchrony. It is a concept that has been appreciated for many years, and was elegantly summarized by Adrian as early as 1935 (Adrian, 1935):

Fortunately, however, the cortex need not be regarded as a system of several million independent units. The cells act in groups, small or large, and sometimes there is a fairly simple co-ordinated wave spreading over a wide area. Activity of this kind occurs only in special conditions, but it gives rise to relatively simple potential changes, and if very many units are involved the potentials are large–large enough to record in man through the unopened skull.

Thus, when cells are synchronously active they are expected to give rise to larger, more easily detectable oscillations. However, this statement needs an immediate clarification. As stated earlier, LFP is thought to arise from the currents generated by active synapses. Thus it would be more accurate to state that large, detectable extracellular oscillations are more likely when *synapses* are synchronously active. Synchronously active synapses are then expected to lead to the synchronous activation (spiking) of local neurons. The historical findings discussed earlier thus reveal the relative synchrony of neurons during different brain states: sleep is characterized by slow delta oscillations reflecting the rhythmic and very synchronous activation of neurons; alert wakefulness is characterized by the so-called “desynchronized” state, where more cells are active overall, but smaller groups are synchronously active. Despite the continued use of the term “desynchronized,” it is perhaps a misnomer. Mercia Steriade was strongly dismissive of the term (Steriade, 1996), referring to those who use it as “epigones” (dictionary definition: “A second rate imitator or follower, especially of an artist or philosopher”). The offense may not be worthy of the term, but Steriade's goal was to point out that active, waking states are associated with oscillations of their own (Steriade and Amzica, 1996). These oscillations may be faster and of lower amplitude, but they are still the result of synchronous and rhythmic activation of cells. We now discuss the rich evidence that has been accumulated over the last few decades linking gamma oscillations with the synchronous activation of cells.

## Gamma-band synchrony: local vs. distant

By 1989 cells in the olfactory system had already been shown to oscillate at gamma frequencies in response to appropriate olfactory cues by Walter Freeman and his colleagues (Freeman, 1978; Di Prisco and Freeman, 1985). Gray and Singer (1989) extended these findings to the visual system. They found that, in response to moving bars of light, the multi-unit activity in cat visual cortex showed synchronous and rhythmic activity at gamma frequencies. This unit activity was accompanied by, and phase locked to, prominent gamma oscillations in the LFP. Later that year, the same group showed that the multi-unit activity at two locations of the visual cortex separated by 2–7 mm could also be synchronous, with almost no phase lag (Gray et al., 1989). Cells that showed such long range synchrony could have very different, non-overlapping receptive fields, but often had almost identical orientation tuning. Contiguous bars of light that spanned both disparate receptive fields resulted in the highest levels of synchrony. In subsequent experiments, similar synchrony with little to no phase lag was seen between regions of V1 and V2 (Engel et al., 1991b), and also across hemispheres (Engel et al., 1991a). Common input, top-down inputs, long-range horizontal corticocortical connections and gap junctions have all been suggested as possible mechanistic explanations for this long-range synchrony, but it remains an area of active research (Engel et al., 2001; Uhlhaas et al., 2009).

These observations have led to what is known as the temporal binding by gamma-band synchrony hypothesis (Singer, 1993). The binding hypothesis states that neurons in anatomically distinct regions can synchronously encode different features of a stimulus (such as color, shape and location) by firing together in a single gamma cycle. This synchronous firing across regions is thought to “bind” the encoded features together into a unified percept of the object, although this theory has proved difficult to test directly (Shadlen and Movshon, 1999). However, there is growing evidence that there are clear behavioral advantages of increased gamma-band synchrony. Recent work suggests that increased gamma-band power is correlated with increased attention (Fries et al., 2001), precedes movement initiation (Pesaran et al., 2002) and is associated with faster reaction times (Womelsdorf et al., 2006).

## Communication via gamma-band synchrony?

What are the mechanistic benefits of synchrony? The exact timing of spikes controls how well information is transmitted from one brain region to another (Abeles, 1982; Softky and Koch, 1993; Konig et al., 1996; Riehle et al., 1997). Synchronous presynaptic inputs can summate more effectively and lead to an action potential in a postsynaptic neuron (Bernander et al., 1994). Thus, when cells in a local brain region spike synchronously during a single gamma cycle, is the information encoded by these cells more likely to be transmitted to downstream brain regions? This is an area of active research. The communication-by-coherency hypothesis, popularized by Pascal Fries and colleagues (Fries, 2009), states that communication between regions that have similar gamma phase is more efficacious than communication between regions that are out of phase (Womelsdorf et al., 2007). However, the amplitude and frequency of gamma oscillations can change rapidly, both spontaneously and in response to changing variables such as visual contrast or running speed (Ray and Maunsell, 2010; Burns et al., 2011; Ahmed and Mehta, 2012). The precise nature of the behavior or stimulus determines whether these gamma frequencies are coherent across different parts of the same brain region. For example, gamma frequencies change together across the hippocampus as a rat runs faster (Ahmed and Mehta, 2012), but different gamma frequencies are observed in different parts of V1 in response to large stimuli with multiple contrasts (Ray and Maunsell, 2010). Thus gamma-band synchrony is only likely to help with the communication of information between regions whose frequency changes coherently. Indeed, a recent study has shown that gamma-band frequencies in V1 and V2 change rapidly but coherently, allowing gamma-band synchrony to play a potentially important role in information transfer between the two regions (Roberts et al., 2013).

## Role of local inhibition in generating gamma

Neurons prefer to fire near the trough of local extracellular gamma oscillations. This finding is remarkably consistent across different species, brain regions and stimuli (Gray and Singer, 1989; Murthy and Fetz, 1992; Bragin et al., 1995; Hasenstaub et al., 2005; Ray et al., 2008; Atallah and Scanziani, 2009; Cardin et al., 2009; Ahmed, 2010; Ray and Maunsell, 2010). Fast-spiking (FS) inhibitory interneurons are thought to play a central role in forcing pyramidal cells to fire in these restricted time windows within gamma cycles. Network models and *in vitro* studies have shown that mutual inhibition between FS interneurons is sufficient to give rise to gamma oscillations (Whittington et al., 1995; Wang and Buzsaki, 1996; Traub et al., 1999; Tiesinga and Sejnowski, 2009). However, there are important differences between the two main FS interneuron-based models of gamma generation, as discussed below.

Theories that propose that interneurons are solely responsible for the generation of gamma are called InterNeuron Gamma models (ING) (Wang and Buzsaki, 1996; White et al., 1998; Traub et al., 1999; Borgers and Kopell, 2003; Borgers et al., 2005; Cardin et al., 2009; Tiesinga and Sejnowski, 2009). In such models, feedback activation of interneurons by pyramidal cells is not necessary: interneurons are driven by external inputs, get inhibited by other interneurons, and then fire again once they have recovered from inhibition. Key parameters that determine the resulting frequency of interneuron firing include the synaptic and membrane time constants, as well as the amount of external excitatory drive of interneurons (Traub et al., 1996). Gap junctions between networks of FS interneurons facilitate their synchronized firing (Traub, 1995; Gibson et al., 1999; Beierlein et al., 2000; Deans et al., 2001), while the mutual inhibition facilitates their gamma rhythmicity. Synchronous, rhythmic interneuron firing then provides periodically inhibited pyramidal cells with a very narrow time window to fire a spike in each cycle, after the previous round of inhibition wears off and just before the next bout of inhibition arrives. Thus inhibition synchronizes the spike times of pyramidal cells, giving rise to gamma-band synchrony among both cell types.

The second class of gamma models is the Pyramidal-InterNeuron Gamma model (PING) (Traub et al., 1999; Borgers et al., 2005; Tiesinga and Sejnowski, 2009). In this model, feedback excitation from pyramidal cells is thought to excite the FS interneurons. The FS interneurons then inhibit the pyramidal cells, and the cycle repeats. This particular class of models does not always include mutual inhibition among FS cells. Which of these two models, if any, provides the most accurate mechanistic description of the neurophysiological mechanisms underlying gamma remains to be fully established (Tiesinga and Sejnowski, 2009).

## Conclusion

As our historical review has shown, the term “desynchronized EEG” was used primarily because larger, slower oscillations such as delta and alpha disappeared when subjects were awake, alert and active. However, as instrumentation improved over the decades, it became clear that even when alpha or other slow oscillations are not present in the waking EEG, there are still faster gamma components (Jasper and Andrews, 1938). As discussed in the second half of this review, there is strong, converging evidence showing that the presence of gamma rhythms in the LFP is accompanied by the synchronous firing of local neurons at the trough of each extracellular gamma cycle, corresponding to the peak of intracellular gamma (Gray and Singer, 1989; Murthy and Fetz, 1992; Bragin et al., 1995; Hasenstaub et al., 2005; Ray et al., 2008; Atallah and Scanziani, 2009; Cardin et al., 2009; Fries, 2009; Tiesinga and Sejnowski, 2009; Adesnik and Scanziani, 2010; Ray and Maunsell, 2010; Buzsaki and Wang, 2012). Thus, given the fact that gamma oscillations are also the result of synchronous activity, the term “desynchronized EEG” is perhaps best permanently replaced by “activated EEG,” as used by Moruzzi and Magoun over 60 years ago (Moruzzi and Magoun, 1949). Unlike the clear local neuronal synchrony during gamma-band oscillations, the extent of long-range gamma-band synchrony and its role in gamma-band communication between areas continues to be worked out and remains an active area of research (Fries, 2009; Ray and Maunsell, 2010; Buzsaki and Wang, 2012; Siegel et al., 2012; Roberts et al., 2013). The high resolution recording techniques and computational power available to today's researchers are a far cry from the simple galvanometers used by Richard Caton and Adolf Beck. But their pioneering work, as well as that of thousands of researchers over the last century, has paved the way toward a better understanding of gamma oscillations. Continuing to work out the detailed mechanisms via which gamma rhythms modulate neuronal synchrony and inter-areal communication promises to help us better understand both the healthy and pathological brain (Fries, 2009; Uhlhaas and Singer, 2010; Buzsaki and Wang, 2012; Siegel et al., 2012).

### Conflict of interest statement

The authors declare that the research was conducted in the absence of any commercial or financial relationships that could be construed as a potential conflict of interest.
